# HIV-V3Augur: A Novel Machine Learning Model for Predicting HIV-1 Tropism in Sub-Subtype A6 and CRF63_02A6, Predominant Variants in Russia and Countries of the Former Soviet Union

**DOI:** 10.3390/v18070703

**Published:** 2026-06-25

**Authors:** Kirill Elfimov, Ludmila Gotfrid, Alina Nokhova, Mariya Gashnikova, Vasiliy Ekushov, Maksim Halikov, Irina Osipova, Dmitriy Baboshko, Andrey Murzin, Ivan Kondeikin, Arina Kiryakina, Aleksey Totmenin, Aleksandr Agaphonov, Natalya Gashnikova

**Affiliations:** 1 State Research Centre of Virology and Biotechnology ‘Vector’, Koltsovo 630559, Russiaand.murzin1@gmail.com (A.M.); ikondeikin@gmail.com (I.K.); kirakinaarina@gmail.com (A.K.);; 2 Federal Research Center for Fundamental and Translational Medicine, Novosibirsk 630060, Russia; alina.nokhova@gmail.com; 3 Department of Natural Sciences, Novosibirsk State University, Novosibirsk 630090, Russia

**Keywords:** HIV-1, tropism, sub-subtype A6, CRF63_02A6, gp120 V3 loop, genotypic tropism prediction, former Soviet Union, Russia

## Abstract

Determining HIV-1 tropism provides the prognosis of HIV infection and is required before prescribing maraviroc, an entry inhibitor that blocks the interaction between the viral gp120 and the CCR5 coreceptor. However, existing prediction algorithms have been developed primarily for the globally most prevalent subtypes (B, C, and CRF01_AE) and often show reduced performance for other HIV-1 genetic variants. Sub-subtype A6 and circulating recombinant form CRF63_02A6 dominate the HIV-1 epidemic in Russia and other Former Soviet Union (FSU) countries, yet the reliability of tropism prediction for these viruses remains virtually unexplored. We phenotypically determined the tropism of 25 clinical isolates (11 R5, 1 X4, and 7 dual-tropic R5/X4) using U87.CD4.CCR5 and U87.CD4.CXCR4 cell lines and performed a comparative analysis of eight existing genotypic tools (Geno2pheno, WebPSSM, T-CUP 2.0, the Delobel/Garrido rules, and others) or their modifications on a combined dataset that included Los Alamos National Laboratory (LANL) reference sequences (subtypes A, B, C, CRF01_AE, and CRF02_AG) and our laboratory-derived isolates. Most models achieved high accuracy for globally prevalent subtypes (≈95% for B, C, and CRF01_AE) but showed markedly reduced performance for sub-subtype A6 (best accuracy among existing models, 85%) and CRF63_02A6 (best accuracy, 72%), with a poor balance between sensitivity and specificity. To address this problem, we developed HIV-V3Augur, an ensemble stacking model based on the Random Forest and Support Vector Machine (SVM) machine learning algorithms, trained on Pseudo Amino Acid Composition (PseAAC) and Relative Synonymous Codon Usage (RSCU) features with 10-fold stratified cross-validation. HIV-V3Augur achieved an accuracy of 77%, sensitivity of 79%, and specificity of 79% on sub-subtype A6, and on CRF63_02A6 it reached an accuracy of 95%, sensitivity of 87%, and specificity of 100%. Cross-validation demonstrated that HIV-V3Augur represents a balanced genotypic tropism prediction tool for understudied HIV-1 variants circulating in the FSU region. HIV-V3Augur can be used locally through a graphical user interface.

## 1. Introduction

HIV-1 entry into the host cell involves sequential interactions of the viral glycoprotein gp120 with the CD4 receptor and one of the chemokine coreceptors, primarily CCR5 or CXCR4 [1]. Viruses that use CCR5 (R5-tropic) are predominantly transmitted and dominate the early stages of infection. Strains that use CXCR4 (X4-tropic) or exhibit dual/mixed tropism (R5/X4) are associated with accelerated depletion of CD4^+^ T-lymphocytes, faster disease progression, and a poorer prognosis [2]. X4 tropism typically emerges at later stages of infection [3]. The only FDA-approved CCR5 antagonist, maraviroc, is effective exclusively against R5-tropic viruses; current clinical guidelines therefore require tropism testing before maraviroc prescription to rule out X4 or R5/X4 variants [4]. Thus, reliable tropism testing is a critical step prior to the initiation of salvage therapy in patients with multidrug resistance, in whom maraviroc is commonly used [5,6].

Phenotypic methods, such as the Trofile assay [7,8], are considered the gold standard for HIV-1 tropism determination [9]. However, they are expensive, labor-intensive, require specialized laboratory facilities [10] and, in some cases, can be performed only at the center that developed the test system [11]. Genotypic methods based on the analysis of the gp120 V3 loop region have become widely used owing to their lower cost, rapid turnaround time, and broader accessibility [12]. Several classes of methods have been developed for genotypic tropism prediction, ranging from simple empirical rules, such as the 11/25 rule and the net charge of the V3 loop [8], to more sophisticated machine learning (ML) algorithms, including support vector machines (SVMs) [13] and Random Forest [14], as well as hidden Markov models [15], neural networks [16], and other approaches. However, the accuracy of these methods varies considerably depending on the HIV-1 genetic variant. The most popular algorithms, such as Geno2pheno and WebPSSM, were developed and trained primarily on sequences of the most prevalent HIV-1 subtype B [17] and subtype C [18]. Several studies have demonstrated that algorithms trained predominantly on subtype B may overestimate or underestimate X4 tropism for non-B subtypes and recombinant forms such as CRF01_AE and CRF02_AG [19,20,21].

The HIV-1 epidemic in Russia and neighboring countries of the former Soviet Union (FSU) is characterized by the predominance of genetic variants other than subtype B. In the European part of Russia, sub-subtype A6 (formerly IDU-A) dominates and accounts for the majority of infections [22]. In the eastern part of Russia (Siberia: Omsk, Novosibirsk, Tomsk, Barnaul), the circulating recombinant form CRF63_02A6 has become predominant and is detected in more than 80% of new HIV-1 cases in this region [23]. Both variants are also widespread in other post-Soviet countries [24,25,26,27], indicating a substantial number of HIV infections caused specifically by these genetic lineages. Despite their epidemiological importance, the evaluation of genotypic tropism prediction tools for these variants has been limited.

Given the increasing prevalence of HIV-1 in Eastern Europe [28] and the use of maraviroc in salvage regimens, there remains a clear need for accurate, validated genotypic tropism determination tools adapted to these genetic variants. The present study focuses on evaluating the accuracy of existing models for genotypic prediction of tropism in sub-subtype A6 and CRF63_02A6 strains. In addition, we present our own software, which offers moderate accuracy for A6 and CRF63_02A6 and can be used by individuals without programming expertise.

## 2. Materials and Methods

### 2.1. Collection of Clinical Isolates and Phenotypic Determination of Tropism

Clinical HIV-1 isolates were obtained from peripheral blood mononuclear cells (PBMCs) of HIV-positive patients from different regions of Russia (Vladivostok, Novosibirsk). Patients were not stratified by treatment experience, treatment failure, or antiretroviral drug resistance (Table 1). This study was approved by the Ethics Committee of the State Budgetary Healthcare Institution “Kraevaya Klinicheskaya Bolnitsa No. 2” (Regional Clinical Hospital No. 2) in Vladivostok (Extract from Meeting Protocol No. 31/1, 10 January 2024). All participants provided informed consent prior to study enrollment.

The HIV-1 isolation procedure included washing PBMCs from whole blood collected in EDTA vacutainers (Greiner Bio-One, Kremsmünster, Austria; Cat. No. 455036) using a Ficoll density gradient (Dia M, Moscow, Russia; Cat. No. Diacoll 1077). Isolated PBMCs from HIV-positive and HIV-negative donors were activated for two days in RPMI-1640 culture medium (Servicebio, Wuhan, China; Cat. No. G4531-500ML) supplemented with 10% fetal bovine serum (FBS) (Thermo Fisher Scientific, Waltham, MA, USA; Cat. No. A5209402) and 5 μg/mL phytohemagglutinin-L (Merck, Darmstadt, Germany; Cat. No. L2646). PBMCs from HIV-positive donors were then co-cultured with PBMCs from HIV-negative donors (at least two donors) in RPMI-1640 medium (Servicebio, Wuhan, China; Cat. No. G4531-500ML) supplemented with 10% FBS (Thermo Fisher Scientific, Waltham, MA, USA; Cat. No. A5209402), 0.001% PenStrep (Thermo Fisher Scientific, Waltham, MA, USA; Cat. No. 15140122), and 0.002% IL-2 (Dia M, Moscow, Russia; Cat. No. PSG210-10). Co-cultivation was carried out for 14 days with monitoring of viral fitness by measuring p24 antigen concentration in the culture supernatant using an HIV-1 p24-antigen-EIA-BEST ELISA kit (VECTOR-BEST, Novosibirsk, Russia; Cat. No. 0134). After virus isolation, the virus-containing supernatant was aliquoted into cryovials (Corning, New York, NY, USA; Cat. No. 431386), frozen at −80 °C for 24 h, and subsequently transferred to liquid nitrogen storage at −196 °C.

Phenotypic determination of tropism was performed using U87.CD4.CCR5 (ARP-4035) and U87.CD4.CXCR4 (ARP-4036) cell lines [29], obtained through the AIDS Reagent Program (currently the Biological and Emerging Infections Research Resources Program, BEI-RRP). Virus-containing culture supernatant was added to the culture medium for both U87 cell lines. The medium consisted of DMEM (Servicebio, Wuhan, China; Cat. No. G4512-500ML) supplemented with 15% FBS (Thermo Fisher Scientific, Waltham, MA, USA; Cat. No. A5209402), 300 μg/mL geneticin (Thermo Fisher Scientific, Waltham, MA, USA; Cat. No. 10131027), and 1 μg/mL puromycin (Servicebio, Wuhan, China; Cat. No. G4017-1ML). Cells were passaged six times, with viral fitness monitored by measuring HIV-1 p24 antigen concentration in the culture supernatant by ELISA using the HIV-1 p24-antigen-EIA-BEST kit (VECTOR-BEST, Novosibirsk, Russia; Cat. No. 0134). A strain was classified as R5-tropic or X4-tropic according to the increase in p24 concentration in the culture supernatant from U87.CD4.CCR5 or U87.CD4.CXCR4 cells, respectively. If an isolate showed an increase in p24 concentration in the supernatant of both cell lines, it was considered a dual-tropic (R5/X4) isolate.

### 2.2. RNA Extraction from Clinical HIV-1 Isolates

Total RNA was extracted from peripheral blood plasma by column-based extraction using the RNA Extraction Kit from Blood Plasma, diaGene (diaGene, Moscow, Russia; Cat. No. 3324.0250). All steps of the procedure were performed according to the manufacturer’s instructions. The obtained RNA samples were stored in a freezer at −80 °C until further analysis.

### 2.3. HIV-1 NFLG Amplification and Sequencing

To obtain near-full-length genome (NFLG) sequences of HIV-1, a two-step nested PCR was performed using custom in-house primers. Complementary DNA (cDNA) was synthesized using the BioMaster RT-PCR-Extra reagent kit (BioLabMix, Novosibirsk, Russia; Cat. No. RM06-200). PCR was carried out with the BioMaster LR HS-PCR kit for long-range DNA amplification (BioLabMix, Novosibirsk, Russia; Cat. No. MH040-400). PCR products were visualized by electrophoresis in a 0.75% agarose gel stained with ethidium bromide.

The resulting PCR amplicons were purified by column-based clean-up using the Cleanup S-Cap kit (Eurogen, Moscow, Russia; Cat. No. BC041L), following the manufacturer’s protocol. After purification, virus-specific fragments were sequenced on the Illumina MiSeq platform (Illumina, Inc., San Diego, CA, USA; Cat. No. SY-410-1003).

### 2.4. HIV-1 Genotyping of Clinical Isolates

Viral genome assembly was performed using BWA v.0.7.17 [30] and iVar v.1.2.2 [31]. The resulting HIV-1 sequences were aligned to reference sequences of various subtypes and recombinant forms available in the international GenBank database, using MEGA11 [32] and AliView v.1.31 [33]. Multiple sequence alignment was performed with MAFFT v.7.526 (RIMD) [34] using standard parameters. A maximum likelihood phylogenetic tree was constructed on the IQ-TREE v.2.4.0 web server with 1000 bootstrap replicates and the GTR + I + G nucleotide substitution model; topological robustness was assessed by bootstrap analysis [35]. The phylogenetic tree was visualized using the Interactive Tree of Life tool (iTOL) [36].

Recombination analysis was performed using the RIP 3.0 web tool [37], available at the Los Alamos National Laboratory (LANL; URL: https://www.hiv.lanl.gov/content/sequence/RIP/RIP.html; accession date: 1 December 2025).

### 2.5. Construction of Reference Sets of HIV-1 gp120 V3 Nucleotide Sequences from LANL

Sequence data for evaluating existing models and for building our own tropism prediction models were retrieved from the LANL HIV nucleotide sequence database (URL: https://www.hiv.lanl.gov/components/sequence/HIV/search/search.html; accession date: 13 April 2026) by filtering for V3 loop sequences with experimentally (phenotypically) confirmed tropism, using the filters “CCR5 only” and “all CXCR4”. We did not separate dual-tropic strains and instead grouped them together with X4-using strains for several reasons: (I) predicting three tropism categories introduces additional complexity for genotypic predictors and reduces their accuracy; (II) many existing models (Geno2pheno [38], PhenoSeq [39], T-CUP 2.0 [40]) do not distinguish between X4 and R5/X4 tropism and assign R5/X4 isolates to the X4-using group; (III) predicting three tropism categories (R5, X4, R5/X4) offers no practical advantage over predicting two categories (R5, X4), because both X4-tropic and R5/X4-tropic HIV-1 strains can use the CXCR4 receptor for cell entry. This precludes the use of maraviroc—the most widely used entry inhibitor.

The most prevalent HIV-1 group M genetic variants, subtypes A, B, and C, as well as the recombinant forms CRF01_AE and CRF02_AG, were selected for the evaluation of existing models and the construction of our own genotypic tropism prediction models. Phenotypically characterized sequences of sub-subtype A6 (n = 19) and CRF63_02A6 (n = 2) were virtually absent from LANL but were used in this study.

The collected sequences varied in length and could include regions of the HIV-1 genome other than the gp120 V3 loop. Therefore, all sequences were uploaded to the GeneCutter web tool (https://www.hiv.lanl.gov/content/sequence/GENE_CUTTER/cutter.html; access date: 14 April 2026) for alignment and extraction of the gp120 V3 region, using standard settings. Other HIV-1 genomic regions were not used for tropism prediction. After the V3 region sequences were obtained, duplicates (sequences with 100% homology) were removed, with one representative sequence retained in each case.

### 2.6. Genotypic Tropism Prediction Methods

**Figure 4:**
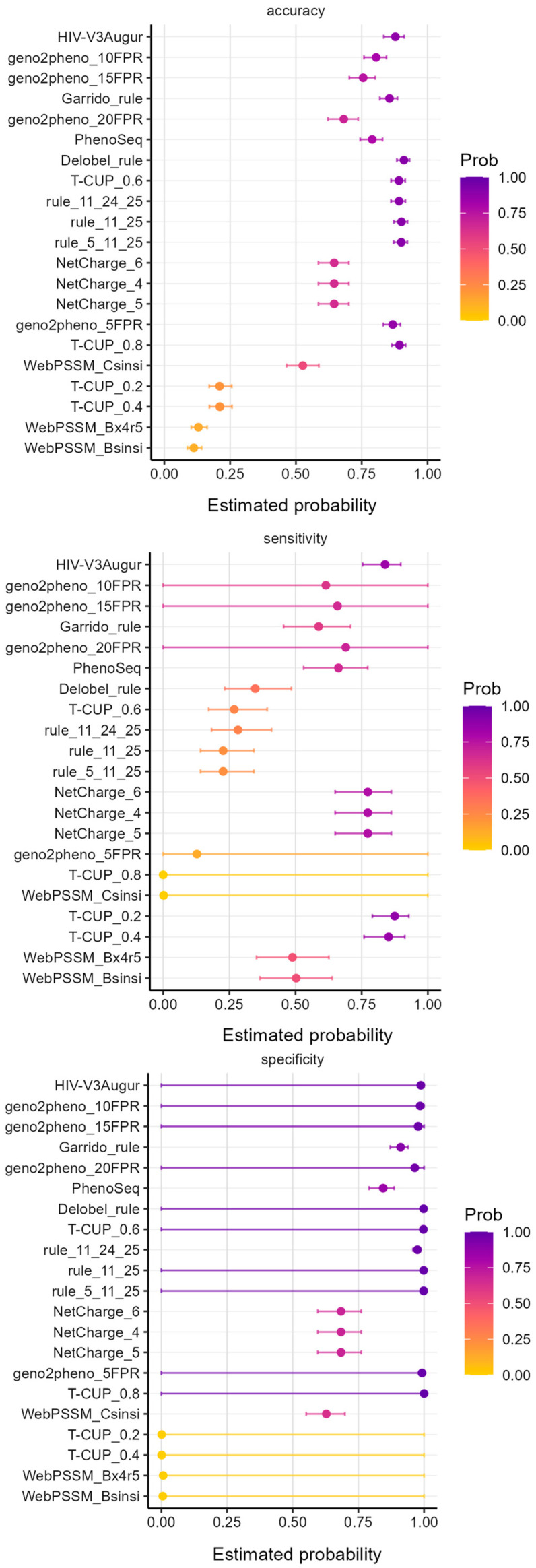
Accuracy, sensitivity (the ability to correctly detect X4 tropism), and specificity (the ability to correctly detect R5 tropism) of models and rules for genotypic tropism prediction of HIV-1 strains based on V3 gp120 sequences. The *Y*-axis shows the models and rules tested; the *X*-axis shows the median metric values (minimum, 0; maximum, 1) with 95% confidence intervals.

We used several existing models (Figure 4) for genotypic tropism prediction of HIV-1 sequences from the LANL database and from our laboratory virus isolates. Some of these models were simple rules: the 11/25 rule, the 11/25/5 rule, the 11/24/25 rule, the net charge rule, and their combinations—the Delobel rule and the Garrido rule. Custom code was written in R v. 4.5.3 (R Core Team, 2024). R: A Language and Environment for Statistical Computing. R Foundation for Statistical Computing, Vienna, Austria; URL: https://www.R-project.org/, access date: 14 April 2026) within the RStudio v. 2026.01.2+418 development environment (RStudio Team, 2019). RStudio: Integrated Development for R. RStudio, Inc., Boston, MA, USA; URL: http://www.rstudio.com/, access date: 14 April 2026) to implement the simple rules.

More complex models were based on ML, for example, on Support Vector Machine (SVM) algorithms (PhenoSeq, HIVcoPRED) or on combinations of several ML algorithms, such as SVM and Decision Tree (Geno2pheno). The T-CUP 2.0 model employed structural modeling of the V3 loop, with calculation of electrostatic potential and hydrophobicity parameters. The WebPSSM model predicted tropism on the basis of a position-specific scoring matrix (PSSM), which consisted of a table of weight coefficients for each amino acid at each V3 position.

### 2.7. Construction of Feature Spaces for Machine Learning

Our own ML models were trained on diverse features calculated from the nucleotide sequences of the HIV-1 gp120 V3 loop: simple amino acid composition (AAC), the amino acid identities at positions 11 and 25 combined with the net charge, Relative Synonymous Codon Usage (RSCU), and pseudo-amino acid composition (PseAAC).

AAC was obtained by translating the V3 nucleotide sequences in MEGA12 software [41].

The dataset containing amino acid identities at positions 11 and 25 along with the net charge values was produced using the custom code written for the simple rule-based tropism prediction methods mentioned above in Section 2.6.

*NetCharge* was calculated as the difference between the number of positively charged amino acids (lysine, arginine) and negatively charged amino acids (aspartate, glutamate) within the V3 amino acid sequence using Formula (1):

(1) $$NetCharge=\left(K+R\right)-\left(D+E\right)$$

where *K*—the number of lysine residues;

*R*—the number of arginine residues;

*D*—the number of aspartate residues;

*E*—the number of glutamate residues.

*RSCU* was calculated using Python v. 3.12.0 (Python Software Foundation. Python Language Reference, version 3.12.0. Available at http://www.python.org, access date: 14 April 2026) with the CodonUsage module from the Biopython v.1.87 package [42], according to Formula (2):

(2) $${RSCU}_{ij=\;}\frac{X_{ij}}{\frac{1}{n_{j}}\;\sum_{k=1}^{n_{i}}X_{ik}}$$

where *i*—the specific amino acid in IUPAC notation;

*j*—the codon of the synonymous group for amino acid *i*;

*X_ij_*—the observed number of the *j* codon for amino acid *i* in the sequence being analyzed;

*n_i_*—the degeneracy of amino acid *i* (the number of distinct codons encoding that amino acid, ranging from 1 to 6);

*k*—the summation index, from 1 to *n_i_*;

∑_(*k* = 1)^(*n_i_*)*X_ik_*—the total count of all synonymous codons encoding amino acid *i*.

PseAAC was also calculated using Python v. 3.12.0 (Python Software Foundation. Python Language Reference, version 3.12.0. Available at http://www.python.org, access date: 14 April 2026), but with the propy3 package [43]. The computation of PseAAC is based on the calculation of AAC and pseudo-components. The pseudo-components (*T_k_*) are calculated according to Formula (3):

(3) $$T_{k}=\;\frac{1}{L-k}\;\sum_{i=1}^{L-k}J_{i,i+k}$$

where *L*—the sequence length;

*k*—the correlation order (the distance between amino acids along the chain for which the interdependence is calculated);

*i*—the starting amino acid position;

*i* + *k*—the index of the amino acid *i* steps away from *k*;

*J*—the similarity function of an amino acid pair (indicating how strongly the physicochemical properties of the amino acids at positions *i* and *i* + *k* differ.

*J* is computed in a separate step using Formula (4):

(4) $$J_{i,j}=\frac{1}{3}\left\{{\left[H_{1}\left(R_{j}\right)-H_{1}\left(R_{i}\right)\right]}^{2}+{\left[H_{2}\left(R_{j}\right)-H_{2}\left(R_{i}\right)\right]}^{2}+{\left[M\left(R_{j}\right)-M\left(R_{i}\right)\right]}^{2}\right\}$$

where *i*—the first amino acid;

*k*—the distance between the two amino acids being compared;

*i* + *k*—the index of the amino acid located *i* steps away from *k*;

*R_i_*—the amino acid residue at position *i*;

*H*_1_—the hydrophobicity on scale 1 (the Tanford scale [44]);

*H*_2_—the hydrophobicity on scale 2 (the Jones scale [45]);

*M*—the side chain mass (measured in Da).

Once the pseudo-component values (*T_k_*) have been computed, the final PseAAC Formula (5) is applied:

(5) $$p_{\left(20+u\right)}=\;\frac{w\times T_{u}}{\sum_{i=1}^{20}f_{i}+w\sum_{k=1}^{\lambda }T_{k}}$$

where *u*—the pseudo-component index;

*p*_(20+*u*)_—the pseudo-component of the amino acid sequence (PseAAC);

*w*—the weighting factor (constant *w* = 0.05);

*T_u_*—the correlation factor of order *u* (*J* from the previous step, normalized by the sequence length (L));

*f_i_*—the frequency of amino acid *i*;

*λ*—the maximum distance at which the interdependence of physicochemical properties is calculated (0 ≤ *λ* < L).

### 2.8. Development of Custom Machine Learning Models

**Figure 1:**
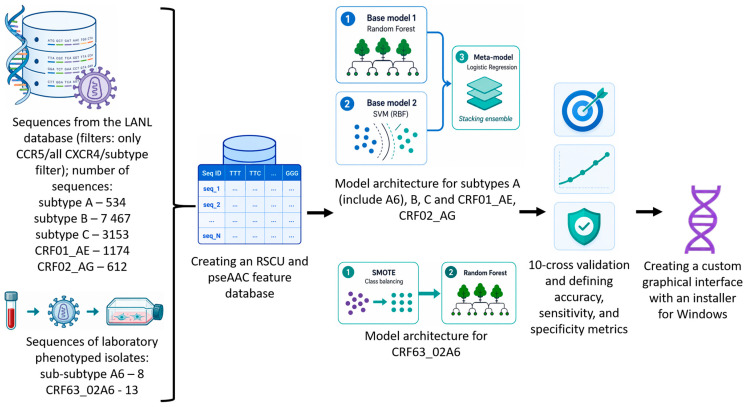
HIV-V3Augur model training workflow. Nucleotide FASTA sequences of the HIV-1 env V3 region were collected for subtype A, subtype B, subtype C, CRF01_AE, and CRF02_AG; CRF02_AG sequences were used as a surrogate training dataset for the CRF63_02A6 classifier. After duplicate removal, exclusion of sequences containing non-canonical nucleotides, and translation, RSCU and PseAAC features were calculated. For subtypes A, B, C, CRF01_AE, and CRF02_AG, subtype-specific stacking models were trained using Balanced Random Forest and SVM as base classifiers and logistic regression as the meta-classifier. For CRF63_02A6, a Random Forest classifier was trained using SMOTE-balanced CRF02_AG data. Model performance was evaluated using stratified 10-fold cross-validation. After 10-fold cross-validation, the models were tested on independent test datasets; laboratory sequences obtained in this study were used for sub-subtype A6 and CRF63_02A6 of HIV-1 (Table 1).

We developed several ML models to predict HIV-1 tropism from V3 loop sequences, all implemented in Python v. 3.12 (Python Software Foundation. Python Language Reference, version 3.12.0. Available at http://www.python.org, access date: 14 April 2026). All computations were performed using the following packages: Biopython v. 1.87 [41], pandas v.3.0.1, numpy v. 2.2.6 [46], scikit-learn v. 1.8.0, imbalanced-learn v.0.14.1 [47], tqdm v. 4.67.3 [48], and joblib v. 1.5.3 (URL: https://joblib.readthedocs.io/en/stable/index.html; access date: 16 April 2026). The complete model training workflow is summarized in Figure 1.

The raw nucleotide sequences of the V3 loop in FASTA format were preprocessed as follows: all sequences were extracted from each file, after which problematic sequences (those containing ambiguous nucleotides) and duplicates (100% homology) were removed, followed by removal of retaining only the first occurrence. For models that used RSCU, the corresponding features were calculated; subsequently, the model was trained and subjected to 10-fold cross-validation. Stop codons were excluded from the analysis.

Tropism prediction for most genetic variants (subtypes A, B, C, CRF01_AE, and CRF02_02AG) employed an ensemble stacking method that combined Random Forest and SVM with a Radial Basis Function (RBF) kernel. Logistic regression served as the meta-classifier, trained on the output probabilities of the base models. To address class imbalance in Random Forest, undersampling of the majority class (R5) was applied at the level of each tree, and in SVM the parameter class_weight = ‘balanced’ was used. For subtype CRF63_02A6, which lacked a sufficient number of sequences for training and cross-validation, a simpler strategy was adopted: sequences from CRF02_AG were used with the Random Forest algorithm and the Synthetic Minority Over-sampling Technique (SMOTE; k = 5). Hyperparameters were selected empirically during preliminary model development; automated grid/random search procedures were explored but were not used in the final training pipeline because they did not improve performance.

All constructed models were evaluated using 10-fold stratified cross-validation. The original data were randomly split into 10 non-overlapping subsets; at each iteration, one subset served as the test set and the remaining nine served as the training set. After ten iterations, the final metrics were calculated as the mean values across all ten folds.

After cross-validation was completed, the following quality metrics were computed for each model: true positives (X4 predicted as X4), false negatives (X4 predicted as R5), true negatives (R5 predicted as R5), and false positives (R5 predicted as X4). Based on these, sensitivity (the proportion of correctly predicted X4), specificity (the proportion of correctly predicted R5), overall accuracy (the proportion of all correct predictions), and the Matthews correlation coefficient (MCC) [49] were calculated [15].

Additionally, the Matthews correlation coefficient (MCC) and the area under the ROC curve (AUC) were evaluated. All metrics were averaged across the 10 folds, with the standard deviation reported. Each trained model was saved in pickle format, and tables with metrics and predictions for all samples were exported.

The complete training workflow, including sequence preprocessing, feature extraction, class-balancing strategy, model architecture, and cross-validation procedure, is summarized in Figure 1. The final empirically selected hyperparameters and training settings for all model components are provided in Supplementary Table S1: Model Training Parameters.

### 2.9. Statistical Analysis

All analyses were performed in R v. 4.4.2 (R Core Team (2024)). Model performance was evaluated for binary classification of viral tropism (R5 vs. X4) across subtypes. Accuracy was defined as the proportion of correct classifications. Sensitivity and specificity were computed with X4 defined as the positive class: sensitivity = P(Ŷ = X4|Y = X4) and specificity = P(Ŷ = R5|Y = R5).

To compare models while accounting for the paired structure of the data, we fitted generalized linear mixed models (GLMMs) with binomial distribution and logit link using lme4:glmer. The fixed-effects structure included model, subtype, and their interaction, and a random intercept was added for each sequence-within-subtype. Separate GLMMs were fitted for (I) accuracy, using all sequences with the binary outcome “correct vs. incorrect”; (II) sensitivity, restricting the dataset to true X4 sequences and modelling “predicted X4 vs. not”; and (III) specificity, restricting the dataset to true R5 sequences and modeling “predicted R5 vs. not”.

Because some model × subtype strata were sparse and exhibited zero-cell patterns (e.g., false positives equal to zero), GLMMs were fitted using a computationally efficient approximation (nAGQ = 0) to ensure stable convergence. Estimated marginal mean probabilities and 95% confidence intervals were obtained on the response scale using the emmeans package. Overall summaries were calculated using equal subtype weights, so that each subtype contributed equally regardless of its sample size. To assess robustness to class imbalance and to reflect a setting where R5 and X4 are assumed to be equally prevalent, we additionally repeated the accuracy analyses using class-balanced weighting (equal total weight for true R5 and true X4 cases within each subtype); sensitivity and specificity were not reweighted because they were estimated conditionally within the true X4 and true R5 subsets, respectively. Pairwise contrasts were reported as differences in response probabilities (Δprob, percentage points) relative to (i) the best-performing model within the relevant context and (ii) the studied model (HIV-V3Augur). *p*-values were adjusted for multiple comparisons using the Holm method, and corrected *p* < 0.05 was considered statistically significant. In some comparisons, Δprob was not reported (shown as “not estimable” in the tables) because sparse subtype strata and zero-cell patterns (e.g., no false positives) resulted in numerically unstable model-based contrasts.

### 2.10. Development of the HIV-V3Augur Graphical User Interface

HIV-V3Augur is implemented in Python 3.12 and distributed as a standalone Windows graphical user interface (GUI) application built with Tkinter v. 8.5 [50]. Sequence parsing, translation, and pairwise alignments are performed with Biopython v.1.87. Data handling and model inference rely on numpy v. 2.2.6, scipy v. 1.17.1 [51] and scikit-learn v. 1.8.0. Pre-trained subtype specific tropism models are stored as pickle (.pkl) objects and loaded at runtime.

Input sequences are provided as nucleotide multi-FASTA files. For each sequence, the V3 region is located by local pairwise alignment of the query nucleotide sequence to a user-supplied V3 nucleotide reference. The extracted V3 nucleotide fragment is then translated to amino acids. Sequences whose extracted V3 contains ambiguous/non ATGC nucleotides are excluded and reported with an exclusion reason.

Genotyping is based on the translated V3 amino acid sequence. Each query V3 AA sequence is compared against curated subtype reference V3 AA sets using global pairwise alignment, and the subtype with the highest similarity is assigned. If multiple subtypes are supported within the predefined similarity margin, the result is reported as an ambiguous multi-candidate call; users can optionally override subtype assignments manually in the GUI.

Tropism is predicted as a binary outcome (R5 vs. X4) using subtype specific pre-trained ML models (.pkl). Ambiguous genotype calls trigger predictions from all candidate subtype models. Outputs include both the predicted class and associated probabilities.

## 3. Results

### 3.1. Characteristics of Clinical Isolates and Phenotypic Determination of Tropism

HIV-positive patients who agreed to provide whole blood were enrolled in this study. All patients provided written informed consent. Patients were not selected based on any clinically relevant parameters, as these parameters were not taken into account in the phenotypic or genotypic determination of tropism. Nonetheless, patients harboring HIV strains of sub-subtype A6 and CRF63_02A6 that caused the infection were specifically recruited. Detailed clinically relevant patient characteristics are presented in the table (Table 1).

Virus isolation was performed according to the standard procedure described in the Collection of Clinical Isolates and Phenotypic Determination of Tropism and the WHO guidelines for the isolation and characterization of HIV strains.

**Figure 2:**
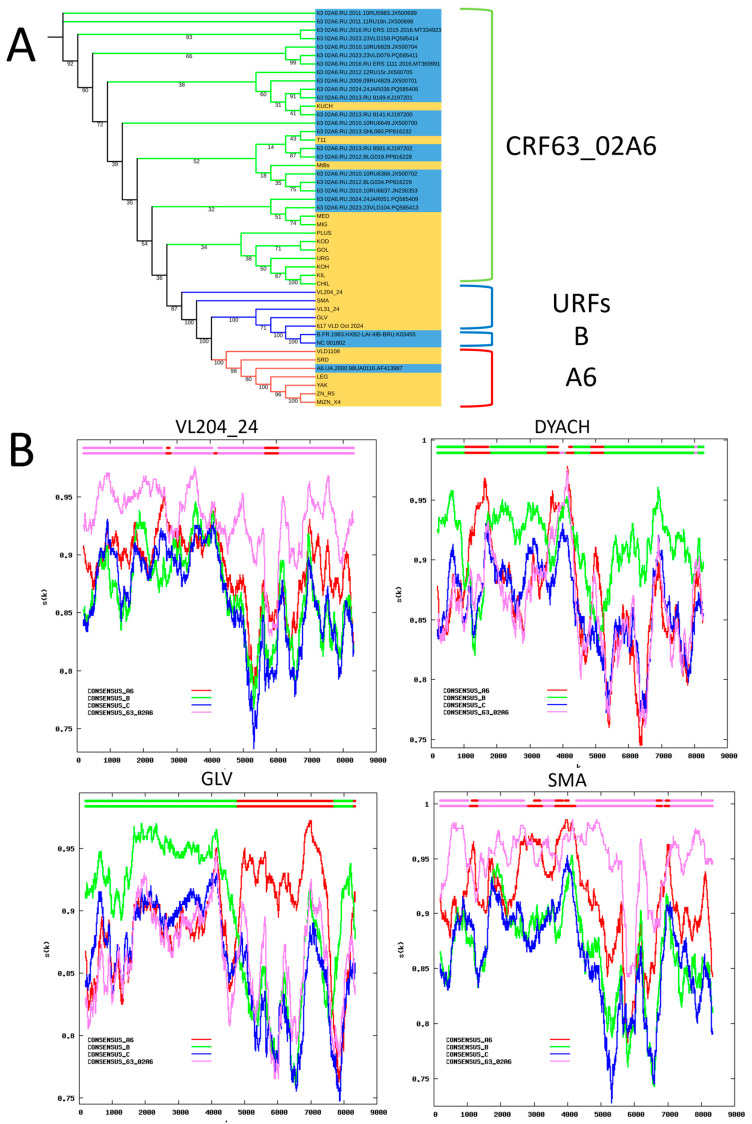
Phylogenetic analysis of laboratory sequences with phenotypically determined tropism: (**A**) Maximum likelihood phylogenetic tree (1000 bootstrap replicates) based on near-full-length HIV-1 genome sequences. Laboratory sequences are shown in yellow, reference sequences in blue. Genotypes are indicated in square brackets. The branch support values are indicated on the branches themselves. (**B**) RIP plots for isolates genotyped as URFs. Colored horizontal bars above each RIP plot indicate genomic regions assigned to different parental genotypes; transitions between colors indicate inferred recombination breakpoints. The V3 region (positions 7092–7310) did not overlap with inferred recombination breakpoints in any URF isolate, allowing each isolate to be assigned to the test set corresponding to the parental variant contributing the V3 region.

Genotyping of the isolates based on near-full-length genome sequences showed that the collected sample was dominated by CRF63_02A6 strains (n = 11), followed by sub-subtype A6 strains (n = 4) and several unique recombinant forms: URF_A6/C/B (n = 1) with the env gene region derived from sub-subtype A6, URF_63/A6 (n = 1) with the env region from CRF63_02A6, and URF_A6/B (n = 2) with the env region from sub-subtype A6. Owing to the scarcity of data, the URFs were assigned to the test sets corresponding to the HIV genetic variant that contributed their env region. Thus, URF_A6/C/B and URF_A6/B were included in the sub-subtype A6 group, and URF_63/A6 was included in the CRF63_02A6 group. We also included several isolates obtained earlier in our laboratory—MTZn (A6; X4-tropic), MTBs (CRF63_02A6; X4-tropic), and T11 (CRF63_02A6; R5-tropic)—as well as a number of phenotypically characterized sequences of sub-subtype A6 (n = 19) and CRF63_02A6 (n = 2) from the LANL database (n = 21; R5 = 18; X4 = 3). All sequences were placed onto a phylogenetic tree (Figure 2). The origin of the V3 region in the URF sequences was analyzed using the RIP tool [37].

**Table 1:** Clinically relevant characteristics of the HIV-positive patients and HIV-1 isolates obtained in this study.

No.	Patient ID	Sex (M = Male, F = Female)	Age (Years)	Disease Stage (Pokrovsky Classification)	Viral Load (HIV RNA Copies/mL Plasma)	CD4^+^ T-lymphocyte Count (Cells/μL)	Transmission Route	Genotype	Phenotypic Tropism	NFLG GenBank Accession Number
1	KUCH	M	43	2B	10 × 10^6^	764	Sexual	CRF63_02A6	R5	PZ426392
2	GOL	M	20	2B	7.2 × 10^6^	433	Sexual	CRF63_02A6	R5/X4	PZ426389
3	KIL	M	32	2B	10 × 10^6^	124	Sexual	CRF63_02A6	R5	PZ426390
4	KOH	F	50	2B	2.67 × 10^6^	268	Sexual	CRF63_02A6	R5	PZ426391
5	PLUS	M	52		3.5 × 10^5^	274	Parenteral	CRF63_02A6	R5	PZ426395
6	MIG	M	29	2B	10 × 10^6^	404	Sexual	CRF63_02A6	R5	PZ426394
7	KOD	F	32	2B	10 × 10^6^	225	Sexual	CRF63_02A6	R5	PX653452
8	VL31_24	M	42		53 × 10^5^			URFA6CB	R5/X4	PZ426402
9	VL204_24	M	51		352 × 10^5^			CRF63_02A6	X4	PZ426403
10	SMA	M	34	2C	10 × 10^6^	1159	Parenteral	URF_63/A6	R5/X4	PX653453
11	YAK	F	40	2B	10 × 10^6^	225	Sexual	A6	R5/X4	PX653454
12	DYACH	M	51		12 × 10^5^	168	Parenteral	URF_A6/B	R5	PZ426400
13	SRD	M	51		256 × 10^5^	534	Sexual	A6	R5	PZ426398
14	CHIL	F	21	2B	10 × 10^6^	319	Sexual	CRF63_02A6	R5	PZ426388
15	URG	F	36	2B	1.5 × 10^6^	702	Sexual	CRF63_02A6	R5	PZ426396
16	MED	F	36	2B	10 × 10^6^	146	Sexual	CRF63_02A6	R5/X4	PZ426393
17	LEG	F	39	2B	10 × 10^6^	229	Sexual	A6	R5	PZ426397
18	GLV	M			620 × 10^5^	148	Parenteral	URF_A6/B	R5/X4	PZ426401
19	VLD1108	F	37		270 × 10^5^	115	Sexual	A6	R5/X4	PZ426399
Summary statistics	n = 19	M = 11 (57.9%) F = 8 (42.1%)	Median = 38; 95% CI: 33.0–46.5	2B: 11/19 (57.9%); 2C: 1/19 (5.3%); NA: 7/19 (36.8%)	Median = 10 × 10^6^; 95% CI: 7.2 × 10^6^–10 × 10^6^	Median = 268; 95% CI: 168–433	Sexual: 13/19 (68.4%); Parenteral: 4/19 (21.1%); NA: 2/19 (10.5%)	CRF63_02A6: 11/19 (57.9%); A6: 4/19 (21.1%); URF_A6/B: 2/19 (10.5%); URF_63/A6: 1/19 (5.3%); URFA6CB: 1/19 (5.3%)	R5: 11/19 (57.9%); R5/X4: 7/19 (36.8%); X4: 1/19 (5.3%)	

For the URF isolates, RIP analysis was used to determine the parental origin of the V3 region of the *env* gene because the V3 loop serves as the genomic target for genotypic tropism prediction. The RIP plots show changes in similarity between each query genome and reference subtype/CRF sequences along the HIV-1 genome. Although several isolates showed recombinant genome structures outside env, the V3 region did not contain inferred recombination breakpoints and was consistently assigned to a single parental lineage. Therefore, these URFs were included in the analysis according to the genetic variant contributing the V3 region: URFs with an A6-derived V3 loop were analyzed with the A6 group, whereas the URF with a CRF63_02A6-derived V3 loop was analyzed with the CRF63_02A6 group. This classification is relevant because the prediction models evaluated in this study use the gp120 V3 loop rather than the whole genome.

**Figure 3:**
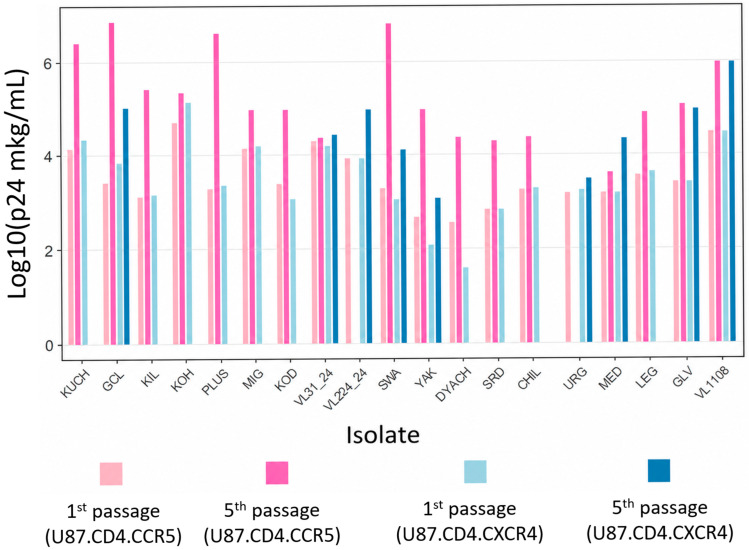
HIV-1 p24 protein concentration (pkg/mL) after the first and fifth passages during cultivation of laboratory isolates on U87.CD4.CCR5 and U87.CD4.CXCR4 cell lines.

Phenotypic tropism determination showed (Figure 3) that the sample contained strains with exclusive R5 tropism (n = 11), exclusive X4 tropism (n = 1), and dual R5/X4 tropism (n = 7).

The evaluation of existing models and the development of our own genotypic tropism prediction models required grouping X4-tropic and R5/X4-tropic strains together as X4-using strains, because most models do not predict dual tropism and introducing three categories would complicate the model, hamper training, and reduce accuracy.

### 3.2. Performance Evaluation of Existing Genotypic Tropism Prediction Models

Eight models and rules were tested in a comparative analysis, including combined rules (Delobel, Garrido), classical rules with modifications (11/25, 11/24/25, 5/11/25), NetCharge (with thresholds of 4, 5, and 6), and more sophisticated genotypic tools (Geno2pheno at FPR thresholds of 5–20%, PhenoSeq, T-CUP with thresholds of 0.2–0.8, and WebPSSM with the Bx4r5, Bsinsi, and Csinsi matrices). The evaluation of common HIV-1 genetic variants (sub-subtypes A1 and A2, subtypes B and C, CRF01_AE, and CRF02_AG) was performed on sequences from the LANL database. The prediction accuracy for sub-subtype A6 was assessed using a combined dataset of LANL and our own sequences, whereas for CRF63_02A6 only laboratory-generated sequences were used.

The Delobel rule achieved the highest overall accuracy (91.13%, 95% CI: 88.45–93.24%), statistically significantly outperforming other simple rules (*p* < 0.05)—the 11/25 rule (90.18%, 95% CI: 87.3–92.47%), the net charge rule (64.69%, 95% CI: 58.69–70.26%)—as well as the more complex models: Geno2pheno at 10% FPR (80.61%, 95% CI: 75.99–84.52%), T-CUP 2.0 at thresholds of 0.2 and 0.4 (21.06%, 95% CI: 17.08–25.69%), PhenoSeq (79.11%, 95% CI: 74.54–83.04%), and WebPSSM with all its variants, for example with the Bsinsi matrix (11.24%, 95% CI: 8.79–14.26%; Figure 4). However, the WebPSSM model was originally developed for tropism prediction of HIV-1 subtype B strains (Bsinsi and Bx4r5 matrices) and subtype C strains (Csinsi matrix), which may explain its low accuracy when predicting tropism for the other subtypes examined here.

Nevertheless, the sensitivity of the Delobel rule remained low (34.45%; 95% CI: 22.84–48.27%), so its high accuracy was primarily driven by its specificity (99.81%; 95% CI: 0–100%), i.e., the ability to detect R5-tropic strains.

The 11/24/25 rule (89.25%, 95% CI: 86.23–91.68%), the 11/25 rule (90.18%, 95% CI: 87.3–92.47%), the 5/11/25 rule (90.12%, 95% CI: 87.22–92.42%), and T-CUP 2.0 with a threshold of 0.6 (89.22%, 95% CI: 86.25–91.61%) all yielded comparable accuracy, with no statistically significant differences (*p* > 0.05). However, all of these models also showed low sensitivity: 27.93% (95% CI: 17.92–40.76%), 22.33% (95% CI: 13.8–34.05%), 22.33% (95% CI: 13.8–34.05%), and 26.5% (95% CI: 16.85–39.08%), respectively.

Sensitivity analysis (the ability to detect X4-tropic variants) revealed a sharp contrast among the models. T-CUP 2.0 with a threshold of 0.2 achieved the highest sensitivity (87.59%; 95% CI: 79.11–92.94%), but its specificity was extremely low (0.12%; 95% CI: 0–100%). T-CUP 2.0 with a threshold of 0.4 also showed high sensitivity (85.30%) and equally low specificity. Among practically viable approaches, the combination of NetCharge with thresholds of 4 and 5 proved the most balanced, with a sensitivity of 77.33% (95% CI: 64.82–86.32%) and a specificity of 68.64% (95% CI: 59.78–76.33%). Geno2pheno at 20% FPR showed high sensitivity (68.93%; 95% CI: 0–100%), but with wide confidence intervals. All classical rules (11/25, 11/24/25, 5/11/25) exhibited extremely low sensitivity (22–28%), making them unsuitable for screening X4 variants.

In terms of specificity (the ability to correctly identify R5-tropic viruses), the best results were predictably yielded by the 11/25 rule (99.84%; 95% CI: 0–100%), the 5/11/25 rule (99.83%; 95% CI: 0–100%), the 11/24/25 rule (97.52%; 95% CI: 95.86–98.53%), and the Delobel rule (99.81%; 95% CI: 0–100%). Geno2pheno maintained specificity above 96% at all FPR thresholds tested (for FPR = 5–99.24%, for FPR = 20–96.51%). In contrast, the models with high sensitivity (T-CUP 2.0 at thresholds of 0.2 and 0.4) exhibited a near-complete loss of specificity.

**Figure 5:**
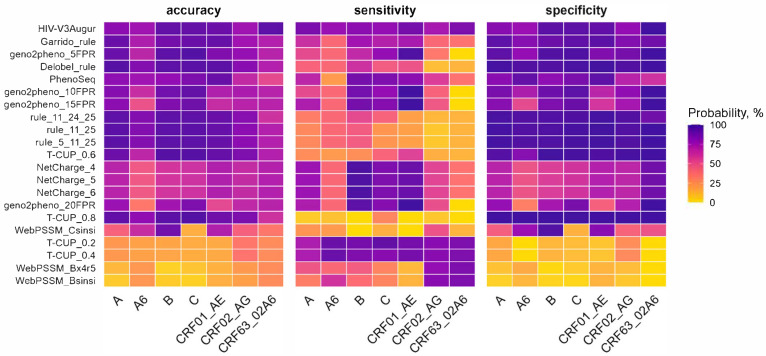
Heatmap of model quality metrics (accuracy, sensitivity, and specificity) for genotypic prediction of HIV-1 tropism based on nucleotide/amino acid sequences of the gp120 V3 loop. Each cell is colored according to the metric value, from yellow (low values, near 0%) to dark blue (high values, near 100%). Data were obtained on sequence subsets for each subtype or sub-subtype (A, A6, B, C, CRF01_AE, CRF02_AG, CRF63_02A6). HIV-V3Augur, our own model, was assessed using 10-fold cross-validation during model training.

When accuracy was examined for individual subtypes and CRFs, many models achieved high accuracy (95–96%) for the globally prevalent variants (B, C, CRF01_AE) (Figure 5).

T-CUP 2.0 with a threshold of 0.6 achieved the highest accuracy on subtype B (96.08%; 95% CI: 94.20–97.37%), followed by T-CUP 0.8 (94.96%; 95% CI: 94.33–97.51%) and the Delobel rule (94.75%; 95% CI: 92.58–96.31%). Geno2pheno at 5% FPR achieved the highest accuracy on subtype C (96.34%, 95% CI: 94.29–97.68%), with T-CUP 2.0 at 0.8 (96.23%; 95% CI: 94.33–97.51%) and T-CUP 2.0 at a threshold of 0.6 (95.73%; 95% CI: 93.72–97.12%) delivering similar results. The Delobel rule performed best on CRF01_AE (95.01%, 95% CI: 92.04–96.91%), followed by T-CUP 0.6 (93.03%; 95% CI: 89.63–95.38%) and the 11/25 and 5/11/25 rules (92.33%; 95% CI: 88.75–94.83%). Meanwhile, the most accurate model for predicting CRF02_AG tropism is T-CUP 2.0, with thresholds of 0.6 and 0.8 showing reduced accuracy (86.41%; 95% CI: 80.62–90.68%) compared with the best models for other common HIV-1 genetic variants. Notably, for CRF63_02A6, several better-performing models (the Delobel rule, Garrido rule, net charge, 11/25 rule, and T-CUP 2.0 with a threshold of 0.6) yielded significantly lower accuracy (71.76%; 95% CI: 38.17–91.28%).

Sensitivity analysis revealed more contrasts. NetCharge (98.03%; 95% CI: 86.35–99.75%) and T-CUP 0.2 (93.88%; 95% CI: 80.42–98.29%) provided the highest sensitivity on subtypes B and C, respectively. Nevertheless, their specificity was very low (Figure 5).

The specificity of the tested models was high for most subtypes (98–100%). However, even the best-performing models in terms of specificity for sub-subtype A6 and CRF63_02A6 (e.g., T-CUP 2.0 with a threshold of 0.8 achieved 100%) did not simultaneously attain acceptable sensitivity. Thus, existing genotypic tools that perform well on the global subtypes B, C, and CRF01_AE show a marked and systematic decline in performance on the genetically distinct variants sub-subtype A6 and CRF63_02A6. None of the tested models were able to achieve both high sensitivity and high specificity for these subtypes, underscoring the urgent need to develop specialized tools for less common but clinically important HIV-1 genetic variants.

### 3.3. Development and Validation of the HIV-V3Augur Model for Genotypic Prediction of HIV-1 Isolate Tropism

We developed a combined stacking model that integrates Random Forest and SVM models to predict HIV-1 tropism from V3 loop nucleotide sequences. The model was trained using 10-fold stratified cross-validation on combined RSCU and pseudo-amino acid composition (PseAAC) data. Logistic regression served as the meta-classifier to improve prediction stability. This ensemble approach ensured reliable discrimination between R5 and X4 tropism for most HIV-1 genetic variants (A, B, C, CRF01_AE, CRF02_AG). Of note, HIV-V3Augur employs distinct models for each of these subtypes to capture the unique patterns of each variant.

**Figure 6:**
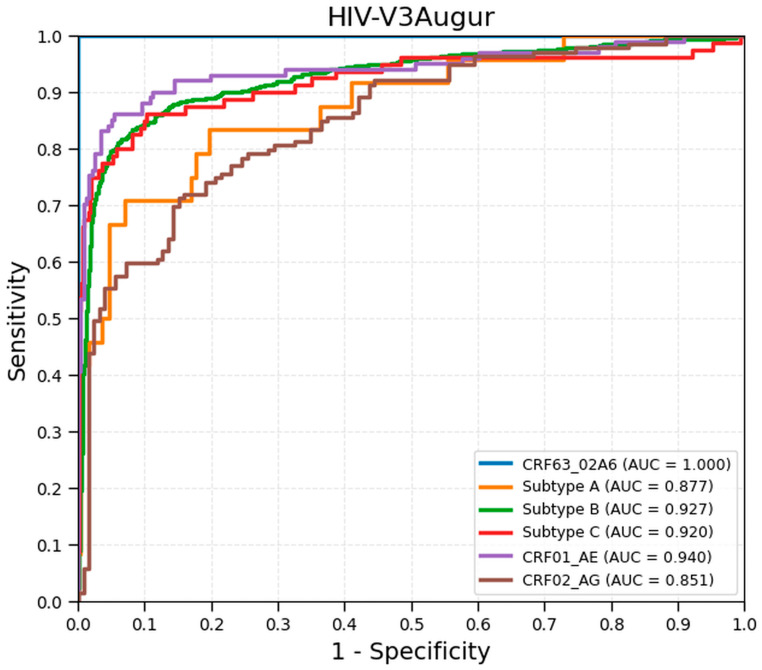
ROC curves of the HIV-V3Augur model for six HIV-1 genetic variants (subtypes A, B, C, CRF01_AE, CRF02_AG, and CRF63_02A6). The area under the ROC curve (AUC) is indicated for each subtype. Colors denote ROC curves for individual subtypes or CRFs. The model demonstrates high discriminatory ability, with AUC values ranging from 0.851 to 1.000. The perfect separation for CRF63_02A6 (AUC = 1.000) should be interpreted with caution, because the sample of phenotyped isolates for this CRF is limited to the present study and there are very few phenotyped sequences in the LANL database. The plot was generated using Python 3.12 with the matplotlib v. 3.10.9, scikit-learn v. 1.8.0, pandas v.3.0.1, and numpy v.2.2.6 libraries.

Notably, the LANL database contains only two CRF63_02A6 sequences with experimentally determined tropism. Direct model training is not feasible due to this limited sample size. Therefore, for the analysis of CRF63_02A6, we used a model trained on the larger and more balanced CRF02_AG dataset. The choice of CRF02_AG as the training set is justified by the fact that, in the CRF63_02A6 genome, the env region, including the gp120 V3 loop, is inherited from this parental variant. The Random Forest algorithm was used for training, with prior synthetic augmentation of the minority (X4-tropic) class by SMOTE. This approach allowed the model to identify X4 tropism signatures that proved applicable to CRF63_02A6 sequences as well. Thus, HIV-V3Augur comprises six distinct classifiers, each tailored to a specific HIV-1 genetic variant. Most classifiers are implemented as a stacking ensemble of Random Forest and SVM, whereas for CRF63_02A6 a separate Random Forest model trained with SMOTE is employed. All models yielded acceptable quality metrics, including accuracy, sensitivity, specificity (Figure 4 and Figure 5), and ROC area (Figure 6). However, the CRF63_02A6 performance estimates should be interpreted cautiously. Because only a very limited number of phenotypically characterized CRF63_02A6 sequences were available, this classifier represents a preliminary surrogate approach based on the parental CRF02_AG env/V3 background rather than a fully independently trained CRF63_02A6-specific model. Therefore, the observed metrics for CRF63_02A6 require confirmation on larger independent datasets.

In the overall evaluation of performance metrics across all subtypes, the HIV-V3Augur model demonstrated balanced performance: median accuracy was 88.3% (95% CI: 84.53–91.9%), sensitivity was 83.29% (95% CI: 73.89–89.77%), and specificity was 98.91% (95% CI: 0–100). HIV-V3Augur ranked sixth in overall accuracy, differing from the top-performing models by only a small margin (0.52% to 2.40%), and these differences were not statistically significant (*p* > 0.05). On the globally prevalent subtypes B, C, and CRF01_AE, however, it was slightly outperformed by the best existing algorithms (e.g., for subtype B, the best accuracy was 96.08% with T-CUP 0.6; for subtype C, 96.34% with Geno2pheno at 5% FPR; for CRF01_AE, 95.01% with the Delobel rule). Nevertheless, our model consistently ranked among the most effective for these variants, confirming its versatility.

The key advantage of HIV-V3Augur became evident when working with the geographically less common subtypes A6 and CRF63_02A6, which together account for the majority of HIV-1 infections in FSU countries. Our model ranked third in accuracy for sub-subtype A6 (77.31%, 95% CI: 55.41–90.33%), surpassed only by the combination of simple rules (Delobel, 11/25, 5/11/25, 11/24/25, which yielded 85.01%). In terms of sensitivity (the ability to detect X4-tropic variants), however, HIV-V3Augur placed second, outperformed only by T-CUP 0.2/0.4 (91.44%), which, in turn, had zero specificity. HIV-V3Augur offered a more balanced and stable profile, with a moderate sensitivity of 79.02% and a specificity of 78.8%. HIV-V3Augur was the absolute leader in accuracy for CRF63_02A6 (94.91%) and also ranked among the best in sensitivity (86.6%) and specificity (100%).

**Figure 7:**
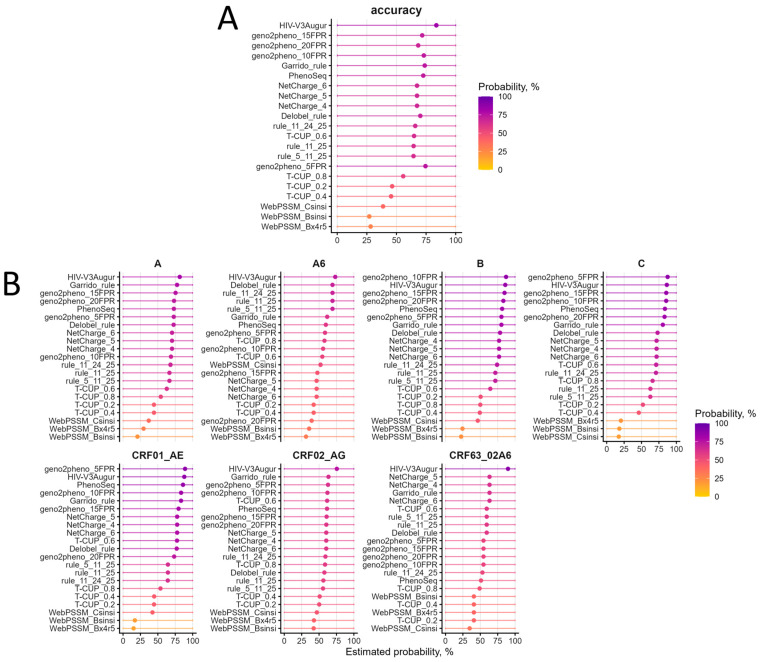
Accuracy of the tested models when analyzing balanced tropism datasets with equal numbers of R5 and X4 sequences: (**A**) median accuracy and 95% CI across all studied HIV-1 subtypes (A, B, C) and CRFs (CRF63_02A6); (**B**) median accuracy and 95% CI of the tested models for each subtype or CRF.

Analysis of the comparative performance of HIV-V3Augur on a balanced dataset containing equal numbers of R5- and X4-tropic strains confirmed the trends of superior performance on sub-subtype A6 and CRF63_02A6 (Figure 7B).

On the balanced dataset (equal numbers of R5 and X4 per subtype), our HIV-V3Augur model yielded the best results for the target subtypes A6 and CRF63_02A6. HIV-V3Augur ranked first in accuracy for sub-subtype A6 (73.24%), outperforming the Delobel, 11/25, and 5/11/25 rules (69.31%). The margin was even wider for CRF63_02A6: HIV-V3Augur reached 89.81%, whereas the second-best model, Garrido, reached only 63.29%.

HIV-V3Augur also led in the averaged assessment across all subtypes, with an overall accuracy of 83.66%, exceeding that of Geno2pheno (74.49%), Garrido (73.85%), and Delobel (70.14%). Thus, with balanced classes, HIV-V3Augur represents an optimal model for genotypic tropism prediction, including for global subtypes.

### 3.4. HIV-V3Augur Graphical User Interface

**Figure 8:**
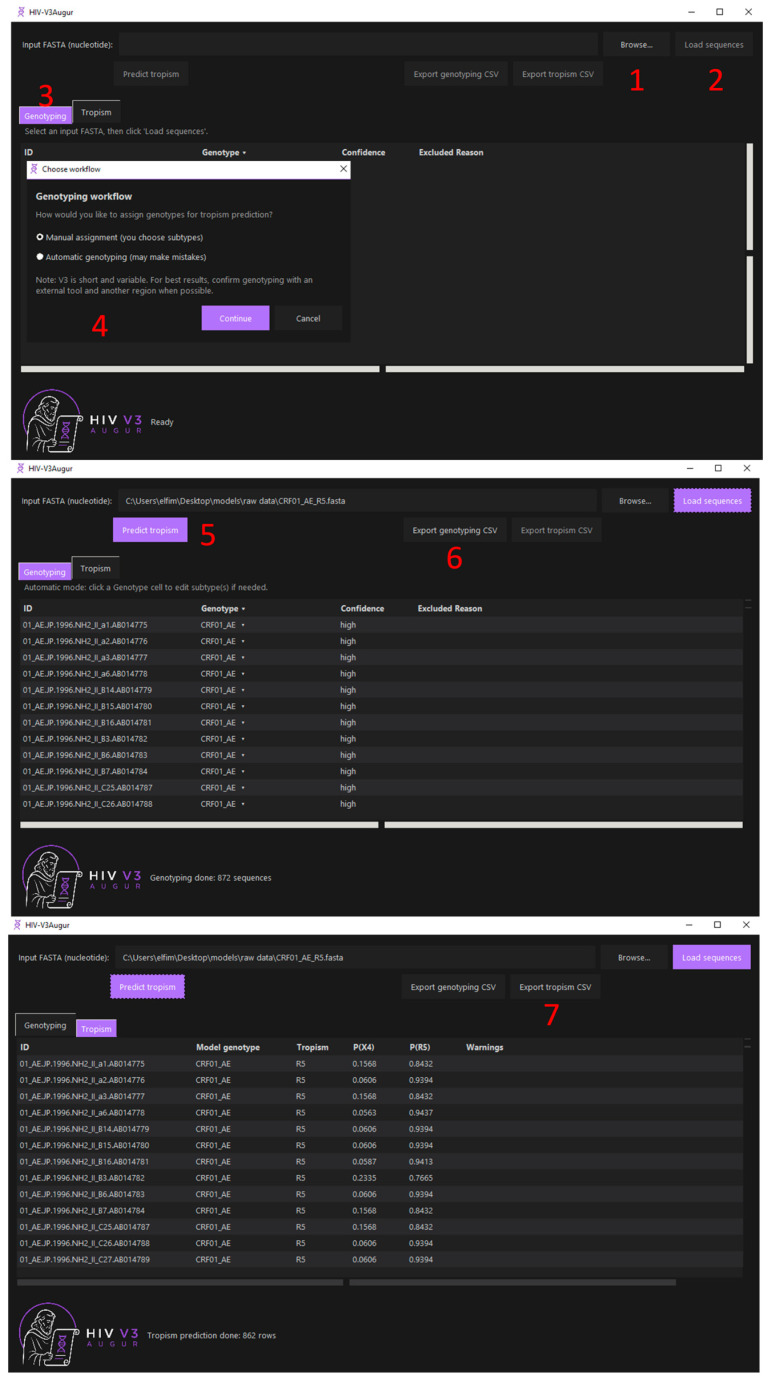
Graphical user interface for the HIV-V3Augur genotypic tropism prediction model. The program allows the user to select a nucleotide FASTA file in any directory (1), load it into the program (2), genotype the isolate sequence manually or automatically (3 and 4), perform genotypic tropism prediction (5), and export genotyping results (6) and tropism predictions (7) as CSV tables.

The developed HIV-V3Augur program is a standalone Windows application with a GUI that requires no installation of additional software or command-line skills. The main window of the program (Figure 8) contains a field for selecting a FASTA file, a button to load the FASTA file, and options for genotyping (automatic or manual; Figure 8). After genotyping is completed, genotype information is displayed. The program can indicate similarity to several genotypes for one sequence and allows the user to select one or multiple genotypes. If the user retains several possible genotypes, HIV-V3Augur predicts tropism for each of them using the corresponding subtype-specific models (Figure 8). The genotyping and tropism prediction results can be saved as a CSV spreadsheet (Figure 8).

When HIV-V3Augur is installed, Windows Security Center may display a warning that the program is untrusted. The program does not collect user data or use the user’s personal computer for any illegal purpose. Users can verify the program’s safety independently, as installed HIV-V3Augur contains code files in its directory.

## 4. Discussion

The present study compared existing genotypic algorithms for predicting HIV-1 tropism on sub-subtype A6 and CRF63_02A6 strains and, in addition, developed and validated a custom ML model, HIV-V3Augur. Phenotypic determination of tropism in the laboratory isolates revealed a predominance of R5-tropic strains (11 of 19 tested), a small number of purely X4-tropic strains (1 of 19), and a substantial proportion of dual-tropic strains (7 of 19). This distribution is consistent with published data: R5-tropic viruses dominate early in infection, and the share of X4 and mixed forms increases with disease progression [52].

Evaluation of the existing genotypic tools (Geno2pheno, WebPSSM, T-CUP 2.0, and others) revealed that the models performed differently depending on the subtype. As expected, algorithms optimized for HIV-1 subtype B demonstrated the highest accuracy on B: for example, the T-CUP 2.0 model with a threshold of 0.6 achieved 96.08% accuracy. The best performer on subtype C was Geno2pheno at FPR5, which attained 96.34% accuracy. However, the accuracy of existing algorithms was markedly lower for sub-subtype A6 and CRF63_02A6. This observation agrees with published data: when applied across a wide range of subtypes and CRFs, genotypic algorithms often show high specificity (correctly identifying R5) but low sensitivity (poor detection of X4) [17]. In particular, Mbondji-Wonje et al. reported that with highly diverse strains, the predictive accuracy for R5 variants was 84–98%, whereas sensitivity for X4 variants reached only 33–50% [53]. Similarly, Mulinge et al. noted that for a number of subtypes and CRFs (e.g., CRF01_AE, CRF02_AG), genotypic algorithms frequently overestimate the proportion of X4-tropic strains and require adaptation to specific variants [20]. Our results likewise demonstrated that at high specificity (low FPR), many algorithms underestimated X4 strains, and at high sensitivity, specificity declined.

Our findings, therefore, underscore the need to develop specialized models for non-B subtypes. Indeed, as noted by Riemenschneider et al., existing algorithms are ineffective for predicting tropism of subtype A and its CRFs, highlighting the necessity of creating new algorithms for genotypic tropism prediction of less common HIV-1 genetic variants [14]. Sub-subtype A6 is a regional variant of subtype A that is, nonetheless, responsible for over a million HIV-1 infections in FSU countries [22]. Our observations from clinical isolates confirm that standard rules (the 11/25 rule, net charge, etc.) and existing algorithms are unbalanced. They exhibit either low specificity or low sensitivity for A6/CRF63, and a more flexible approach is therefore needed.

The proposed HIV-V3Augur model, which is based on machine learning with Random Forest and SVM algorithms, showed balanced performance metrics across multiple HIV-1 genetic variants (A, B, C, CRF01_AE, CRF02_AG, CRF63_02A6). During validation, it achieved an AUC > 0.85 (Figure 5), along with an accuracy of 88.73%, sensitivity of 83.29%, and specificity of 98.91% on a combined test set comprising all studied HIV-1 genetic variants. Importantly, HIV-V3Augur demonstrated high accuracy for sub-subtype A6 and CRF63_02A6: 77.31% and 94.91%, respectively.

Beyond predictive performance, the biological interpretation of the feature space is important. In HIV-V3Augur, RSCU features capture codon-usage patterns within the V3 nucleotide sequence, whereas PseAAC features summarize amino acid composition together with sequence-order and physicochemical properties of the translated V3 peptide. These descriptors may reflect different biological layers of the same tropism-associated region: nucleotide-level evolutionary constraints, subtype-specific codon usage, amino acid composition, charge, hydrophobicity, and local physicochemical context [54,55,56]. Importantly, these features should not be interpreted as direct mechanistic determinants of tropism by themselves; rather, they provide machine learning descriptors that may capture subtype-specific sequence patterns associated with experimentally determined CCR5 or CXCR4 usage [7,57].

The clinical consequences of incorrect tropism prediction are also asymmetric. A false R5 prediction for an X4-using or dual/mixed virus is the most clinically concerning error, because it may incorrectly justify CCR5 antagonist therapy in the presence of CXCR4-using virus [8,58]. Maraviroc is indicated only for CCR5-tropic HIV-1 and is not recommended for dual/mixed- or CXCR4-tropic infection [59,60]. Therefore, insufficient sensitivity for X4-using variants may lead to inappropriate treatment selection and possible virological failure.

A false R5 prediction may also allow CCR5 antagonist therapy in the presence of pre-existing CXCR4-using or dual/mixed variants [61,62]. In this context, suppression of CCR5-tropic viruses could theoretically unmask or favor the relative outgrowth of CXCR4-using populations, particularly if the accompanying background regimen is insufficiently suppressive [63]. This is clinically relevant because CXCR4-using variants have been associated with faster disease progression and may preferentially involve naive and central memory CD4+ T-cells [64]. However, this risk should be interpreted cautiously, since clinical and modeling data suggest that maraviroc combined with potent background therapy does not necessarily promote de novo X4 emergence.

Conversely, a false X4 prediction for a truly R5-tropic virus would not expose the patient to an ineffective CCR5 antagonist, but it may unnecessarily exclude maraviroc from the treatment options, which is particularly relevant for treatment-experienced patients with limited antiretroviral choices [65]. Thus, an optimal genotypic tropism model should not maximize accuracy alone but should maintain a clinically acceptable balance between sensitivity for X4-using viruses and specificity for R5 viruses [66].

The clinical consequences of incorrect tropism prediction are also asymmetric. A false R5 prediction for an X4-using or dual/mixed virus is the most clinically concerning error, because it may incorrectly justify CCR5 antagonist therapy in presence of CXCR4-using virus. Maraviroc is indicated only for CCR5-tropic HIV-1 and is not recommended for dual/mixed- or CXCR4-tropic infection. Therefore, insufficient sensitivity for X4-using variants may lead to inappropriate treatment selection and possible virological failure. Conversely, a false X4 prediction for a truly R5-tropic virus would not expose the patient to an ineffective CCR5 antagonist, but it may unnecessarily exclude maraviroc from the treatment options, which is particularly relevant for treatment-experienced patients with limited antiretroviral choices. Thus, an optimal genotypic tropism model should not maximize accuracy alone but should maintain a clinically acceptable balance between sensitivity for X4-using viruses and specificity for R5 viruses.

Several limitations of the present work should be noted. First, the clinical sample size was relatively small (a few dozen A6/CRF63_02A6 isolates), and the number of available sequences for these variants from LANL was extremely limited. The limited number of phenotypically characterized A6 and especially CRF63_02A6 sequences may affect the robustness of the estimated model performance. Small datasets increase the variance of accuracy, sensitivity, specificity, MCC, and AUC estimates, and individual misclassified sequences can have a disproportionately large effect on the final metrics. This limitation is particularly important for X4-using viruses, which were less frequent than R5 viruses, and for CRF63_02A6, for which direct subtype-specific training was not feasible because only a very small number of phenotyped sequences were available. As a result, the CRF63_02A6 classifier should be interpreted as a preliminary surrogate approach based on the parental CRF02_AG V3 region background rather than as a fully independently trained CRF63_02A6-specific model. Previous reports have already emphasized the need for further algorithm refinement and large-scale studies with a greater number of diverse strains and sequences [53]. Future work should prioritize multicenter collection, phenotypic characterization, and sequencing of additional A6 and CRF63_02A6 isolates. As these data become available, HIV-V3Augur should be retrained and externally validated to improve model stability, refine subtype-specific decision boundaries, and better estimate performance across clinically relevant R5, X4, and dual R5/X4 variants.

Second, the model relies on the analysis of the V3 loop, whereas evidence has been accumulating that other regions of the *env* gene, as well as *tat* and *nef* genes, also influence tropism determination [67,68,69,70].

Nevertheless, the results obtained demonstrate that HIV-V3Augur is a promising model for genotypic tropism prediction. As new data become available, the models can be retrained to refine HIV-V3Augur and improve its quality metrics. In a broader context, the ML-based approach demonstrates that accounting for regionally dominant HIV variants can improve the accuracy of the analysis, which is relevant for many less common subtypes and CRFs worldwide.

Promising directions for future research include isolation, phenotyping, and sequencing of a larger number of A6/CRF63_02A6 strains through the coordinated efforts of several laboratories in FSU countries, as well as improving genotypic tropism prediction approaches by incorporating information from regions outside the V3 env region.

## 5. Conclusions

This study demonstrates that existing genotypic algorithms for HIV-1 tropism prediction, which were developed primarily based on subtypes B and C, show a marked decline in performance when applied to the genetic variants dominant in Russia and other post-Soviet countries—sub-subtype A6 and the recombinant form CRF63_02A6. Even the best-performing tested models (the Delobel rule, T-CUP 2.0, and Geno2pheno) were unable to provide balanced sensitivity and specificity for these variants, exhibiting either unacceptably low sensitivity for X4-tropic strains or low specificity. These findings highlight the urgent need to develop specialized tools for regionally relevant HIV-1 genetic variants.

To address this issue, we developed HIV-V3Augur, a combined ensemble classifier built on the Random Forest and SVM machine learning algorithms and trained on balanced feature sets (PseAAC, RSCU) with 10-fold stratified cross-validation. Because phenotyped sequences for CRF63_02A6 were extremely rare, we applied a model trained on the parental CRF02_AG, which achieved high accuracy (94.9%) while maintaining sensitivity (86.6%) and specificity (100%). HIV-V3Augur provides more balanced accuracy, sensitivity, and specificity for sub-subtype A6 and CRF63_02A6 compared with other genotypic tropism prediction models for HIV-1. The model is integrated into a graphical user interface, making it accessible to laboratories without specialized bioinformatics expertise.

Nevertheless, because phenotypically characterized A6 and especially CRF63_02A6 sequences remain limited, the current model should be regarded as a preliminary subtype-informed framework for which it is desirable to carry out further training and validation with the cooperation of several laboratories in the countries of the FSU in order to obtain a larger number of phenotyped HIV-1 isolates.

## Data Availability

The nucleotide sequences of the isolates are available in the open GenBank database under accession numbers PX653452-PX653454 and PZ426388-PZ426403. HIV-V3Augur is available on a Zenodo repository (DOI: 10.5281/zenodo.20196437; URL: https://zenodo.org/records/20196438 (access date: 15 May 2026)).

## Supplementary Materials

The following supporting information can be downloaded at: https://www.mdpi.com/article/10.3390/v18070703/s1, Table S1: Model Training Parameters.

## Abbreviations

The following abbreviations are used in this manuscript:

AAC	Amino Acid Composition
AIDS	Acquired Immunodeficiency Syndrome
AUC	Area Under the ROC Curve
CCR5	C-C chemokine receptor type 5
cDNA	Complementary DNA
CRF	Circulating Recombinant Form
CXCR4	C-X-C chemokine receptor type 4
ELISA	Enzyme-Linked Immunosorbent Assay
FBS	Fetal Bovine Serum
FDA	Food and Drug Administration
FPR	False Positive Rate
FSU	Former Soviet Union
GLMM	Generalized Linear Mixed Model
GUI	Graphical User Interface
HIV	Human Immunodeficiency Virus
LANL	Los Alamos National Laboratory
ML	Machine Learning
NFLG	Near-Full-Length Genome
PBMC	Peripheral Blood Mononuclear Cells
PseAAC	Pseudo-Amino Acid Composition
PSSM	Position-Specific Scoring Matrix
RIP	Recombinant Identification Program
RBF	Radial Basis Function
ROC	Receiver Operating Characteristic
RSCU	Relative Synonymous Codon Usage
SMOTE	Synthetic Minority Over-sampling Technique
SVM	Support Vector Machine
URF	Unique Recombinant Form
